# Systemic Inflammatory Reaction in Patients With Head and Neck Cancer—An Explorative Study

**DOI:** 10.3389/fonc.2019.01177

**Published:** 2019-11-05

**Authors:** Thorsteinn Astradsson, Felix Sellberg, David Berglund, Ylva Tiblom Ehrsson, Göran Frans Emanuel Laurell

**Affiliations:** ^1^Department of Surgical Sciences, Uppsala University, Uppsala, Sweden; ^2^Department of Immunology, Genetics and Pathology, Uppsala University, Uppsala, Sweden

**Keywords:** radiotherapy, chemoradiotherapy, cisplatin, cytokines, immune system, growth factors

## Abstract

**Aim:** To assess the longitudinal pattern of pro-inflammatory cytokines and growth factors in serum up to 1 year following treatment for head and neck cancer.

**Materials and Methods:** Patients with newly diagnosed, curable head and neck cancer were included (*n* = 30). The most common subsite was oropharynx (*n* = 13) followed by oral cavity (*n* = 9). Blood was drawn from all patients at regular intervals (before treatment, 7 weeks after the start of the treatment, and at 3 months and 1 year after termination of treatment) and analyzed for cytokines (Il-1β, Il-2, Il-4, Il-5, Il-6, Il-8, Il-10, GM-CSF, TNF-α, and IFN-γ) and growth factors (G-CSF, FGF-2, EGF, and VEGF).

**Results:** The time point of the peak level of pro-inflammatory cytokines was 7 weeks after start of treatment which corresponded for the majority of patients with termination of radiotherapy or chemoradiotherapy. Patients undergoing chemoradiotherapy exhibited a significant increase of IL-1β, IL-6, and IL-10 at 7 weeks as compared to pre-treatment levels. At 1 year after termination of treatment four patients experienced recurrence of disease while 26 patients were considered disease-free. The patients with recurrence had significantly higher levels of IL-1β, IL-6, IL-8, and IL-10 at 7 weeks after the start of treatment than patients without recurrence. Correlated with T stadium patients with T3-T4 had higher levels of IL-1β and IL-8 than patients with T1-T2 7 weeks after the start of treatment.

**Conclusions:** The observed immune response in this explorative study demonstrates that chemoradiotherapy may induce not only a local treatment effect on the immune system but also effects far outside the irradiated field. The result of the study indicates that analysis of a pro-inflammatory panel of cytokines in serum at 7 weeks after the start of treatment could be of prognostic value in patients with head and neck cancer. Further study of a larger cohort could help identify patients at larger risk for recurrent disease with measurements of pro-inflammatory cytokines under and after treatment.

## Introduction

Over the last decade knowledge in cancer immunology has grown impressively. Its clinical application in the management of head and neck cancer is still rather poorly explored but the growth of scientific publications is increasing. Head and neck cancer constitutes a heterogeneous group of cancers, the majority with an epithelial origin, that have been characterized as immunosuppressive malignancies (1). A mainstay of head and neck cancer treatment is external beam radiotherapy which is known to have the capacity to induce a local and systemic immune response (2). This complex immune response is regulated among others by pro-inflammatory cytokines in the so-called inflammasomes, cytosolic multiprotein complexes, which have been associated with both carcinogenesis and treatment response in head and neck cancer (3, 4). At a local level the immune response is known to give rise to a cytotoxic effect and eradicate tumor cells, but at a systemic level the immune system can contribute to increased side effects. The local immune reaction also gives rise to several side effects. Chemoradiotherapy is now the gold standard for advanced cancer in several sites in the upper aerodigestive tract. Data about the immune response induced by chemoradiation is scarce in clinical trials.

Pro-inflammatory cytokines and growth factors are involved in a wide range of functions in inflammation, immune response, tumor microenvironment, and homeostasis. Pro-inflammatory cytokines interleukin-1 beta (IL-1β), interleukin 6 (IL-6), and interleukin 8 (IL-8) are mediators of acute phase response, which is often associated with induction of an oxidative stress situation (5, 6). Their multifactorial functions have been described in several organ systems and diseases. Besides a role in the immune reaction, IL-6 is reported to have metabolic functions (7). Pro-inflammatory cytokine secretion in the blood is associated with an immune response that can be seen in some patients with head and neck cancer before treatment is initiated (8). Patients with the highest levels of pro-inflammatory cytokines were reported to have the poorest prognosis. In another study on patients with oral cancer, the highest levels of IL-6 in cancer tissue were found to be associated with distant metastasis (9). IL-1β, IL-6, and tumor necrosis factor alpha (TNF-α) can be used as biomarkers of inflammation during radiotherapy and chemoradiotherapy, and their role in head and neck cancer treatment needs to be better explored as pro-inflammatory cytokines activated by radiotherapy may increase the symptoms of cancer treatment (10, 11). Adaptive immune response regulation is driven by cytokine profiles associated with subsets of CD4^+^ T-cells. Cytokines interleukin-2 (IL-2), interferon gamma (IFN-γ), and TNF-α are associated with a cytotoxic T_H_1 response while cytokines such as interleukin-4 (IL-4) and interleukin-5 (IL-5) are associated with a humoral T_H_2 response (12). The T_H_1 response has been correlated to an improved clinical outcome and associated cytokines have been investigated as a therapy to cancer (13). Interleukin-10 (IL-10) is immunosuppressive in nature preventing the production of T_H_1 cytokines (such as IL-2, IFN-γ, and TNF-α), increased circulating levels have been found in several types of cancer including oral squamous cell carcinoma and are correlated to a negative prognosis (14, 15). Moreover, the growth factors epidermal growth factor (EGF) and vascular endothelial growth factor (VEGF) can eventually give additional information as genetic variants of these biomarkers have shown association to treatment response in esophageal squamous cell carcinoma (16).

More extensive profiles of pro-inflammatory cytokines and growth factors in serum have not been previously studied longitudinally in patients undergoing head and neck cancer treatment. Information drawn from a study where these biomarkers are assessed in detail and correlated with recurrence could possibly provide valuable information which could help to identify patients at greater risk for the need of nutritional support and even recurrent disease. Here we present an explorative study on an unselected cohort of patients with head and neck cancer. The main aim of this prospective study was to assess the longitudinal pattern of pro-inflammatory cytokines and growth factors in serum up to 1 year following treatment.

## Materials and Methods

### Study Design

The study was a prospective explorative study performed at three head and neck cancer centers in Sweden. Inclusion criteria were curable newly diagnosed untreated head and neck cancer and a performance status of 0–2 according to the WHO. Exclusion criteria included previous treatment for malignant neoplasms within the last 5 years except for skin cancer, severe alcoholism, dementia, or other inability to participate and inability to understand Swedish. The Regional Ethical Review Board in Uppsala reviewed and approved the study (No. 2014/447). Blood samples were coded and stored in the Uppsala Biobank (approved RCC 2015-0025). The study is registered at ClinicalTrials.gov, NCT03343236.

### Study Subjects

All patients were under nutritional surveillance according to local routines and were offered nutritional supplemental therapy when indicated. All patients were staged according to UICC 7 staging system. A study representative met with included patients before treatment, at 7 weeks after the start of treatment and at 3 months and 1 year after the termination of treatment. Weight was followed up at each visit. Height was measured and used for the calculation of BMI (kg/m^2^). The study cohort consisted of 30 patients undergoing treatment for head and neck cancer. The mean age was 66 years (range 50–85) and the male-to-female ratio of 2.3:1 (21 males, 9 females). The most common subsite was oropharynx (*n* = 13) followed by the oral cavity (*n* = 9). Of the patients with oropharyngeal cancer, 12 patients were p16 positive while one patient had a p16 negative tumor. Treatment consisted of surgery alone, radiotherapy alone, surgery and radiotherapy with or without chemotherapy, and chemoradiotherapy alone. Altogether 10 patients received chemoradiotherapy, nine of these patients were given cisplatin-based chemotherapy. Patient characteristics are summarized in Table 1.

**Table 1:** Characteristics of studied patients in cohort treated for head and neck cancer.

**Patient**	**Localization**	**T**	**N**	**M**	**Stage**	**Treatment**	**Type of chemotherapy/ monoclonal antibody**
1	Oral	T2	N1	M0	III	Surgery and adj CRT	Cisplatin
2	Oral	T2	N0	M0	II	Surgery and adj RT	
3	Hypopharynx	T4	N2C	M0	IVA	CRT	Cetuximab
4	Oral	T2	N0	M0	II	Surgery and adj RT	
5	Oropharynx	T4	N2B	M0	IVA	CRT	Cisplatin
6	Larynx	T1	N0	M0	I	RT	
7	Larynx	T2	N0	M0	II	Neo-adj RT and surgery	
8	Oral	T2	N2C	M0	IVA	Surgery and adj RT	
9	Oropharynx	T3	N2B	M0	IVA	CRT	Cisplatin
10	Oral	T2	N0	M0	II	Surgery and adj RT	
11	Oropharynx	T1	N1	M0	III	CRT	Cisplatin
12	Larynx	T1	N0	M0	I	Surgery	
13	Oral	T2	N1	M0	III	Surgery and adj RT	
14	Oropharynx	T1	N2C	M0	IVA	CRT	Cisplatin
15	Oropharynx	T2	N1	M0	III	RT	
16	Other^*^	X	N0	M0	X	Surgery	
17	Oropharynx	T1	N1	M0	III	Surgery and adj CRT	Cisplatin
18	Oropharynx	T2	N2B	M0	IVA	RT	
19	Larynx	T2	N0	M0	II	RT	
20	Oropharynx	T4	N2B	M0	IVA	CRT	Cisplatin
21	Oropharynx	T2	N0	M0	II	RT	
22	Oropharynx	T2	N2B	M0	IVA	RT	
23	Oropharynx	T2	N2B	M0	IVA	RT	
24	Oral	T1	N0	M0	I	Surgery	
25	Oral	T1	N2B	M0	IVA	Surgery and adj CRT	Cisplatin
26	Oropharynx	T4	N2B	M0	IVA	RT	
27	CUP	T0	N2B	M0		Neo-adj RT and surgery	
28	Larynx	T1	N0	M0	I	RT	
29	Oropharynx	T4	N2B	M0	IVA	CRT	Cisplatin
30	Oral	T1	N0	M0	I	RT	

*CRT, Chemoradiotherapy; RT, Radiotherapy; Adj, Adjuvant; CUP, Cancer of Unknown Primary*;

* *Adenoid cystic cancer of external auditory canal. TNM and stage according to UICC version 7*.

### Analysis of Cytokines and Growth Factors

Blood was drawn from all patients at regular intervals (before treatment, 7 weeks after the start of the treatment, and at 3 months and 1 year after termination of treatment) and stored as serum at −70°C at the Uppsala University Hospital Biobank, Uppsala. The serum was later analyzed for cytokines (Il-1β, Il-2, Il-4, Il-5, Il-6, Il-8, Il-10, granulocyte-macrophage-colony-stimulating factor (GM-CSF), TNF-α, and IFN-γ) using the 10-plex ultra-sensitive cytokine assay (Thermo Fisher Scientific, Waltham, MA, US) and additional factors granulocyte colony stimulating factor (G-CSF), fibroblast growth factor 2 (FGF-2), EGF, and VEGF using the Luminex xMAP technology (Merck Millipore, Darmstadt, Germany). Both using 50 μl of centrifuged clarified serum (2,000 g × 10 min) as a sample. The samples were analyzed on a Luminex 100/200 system (Luminex Corporation, Austin, TX, US) running the xPONENT software (Luminex Corporation). Standard curves were used to interpolate values using five parameter logistics. Values falling below the analytical sensitivity were defined as 1.6 μg/ml (half the detection limit) for G-CSF, EGF, FGF-2, and VEGF and as 0 μg/ml for the ultra-sensitive cytokine assay.

### Statistical Analysis

After the analysis was performed residuals were kept and tested for normality using the Shapiro–Wilks test. Data were not normally distributed, so none-parametric tests were used when possible. When testing the change over time Friedman's test was used and if found significant follow-up testing was done using Dunn's multiple comparison test (with correction for multiple analyses). When observing the changes over time patients were stratified on treatment modality, T-stage and recurrence of disease, respectively, a two-way ANOVA model was used comparing the groups with Tukey's multiple comparisons test (with correction for multiple analyses).

All graphs are presented with mean values ± SEM, statistically significant differences are shown and denoted with a ^*^. The level for statistical significance was set to *p* = ≤ 0.05.

## Results

Tumor response to treatment and loco-regional status were regularly assessed by physicians at each center participating in the study by clinical examination. When indicated, response to treatment was also assessed with CT or ^18^F-FDG-PET/CT. At 1 year after termination of treatment 26 patients were considered disease-free and four patients had documented recurrence. Of the four failures, three patients were diagnosed with loco-regional recurrence and one patient with generalized disease.

**Figure 1:**
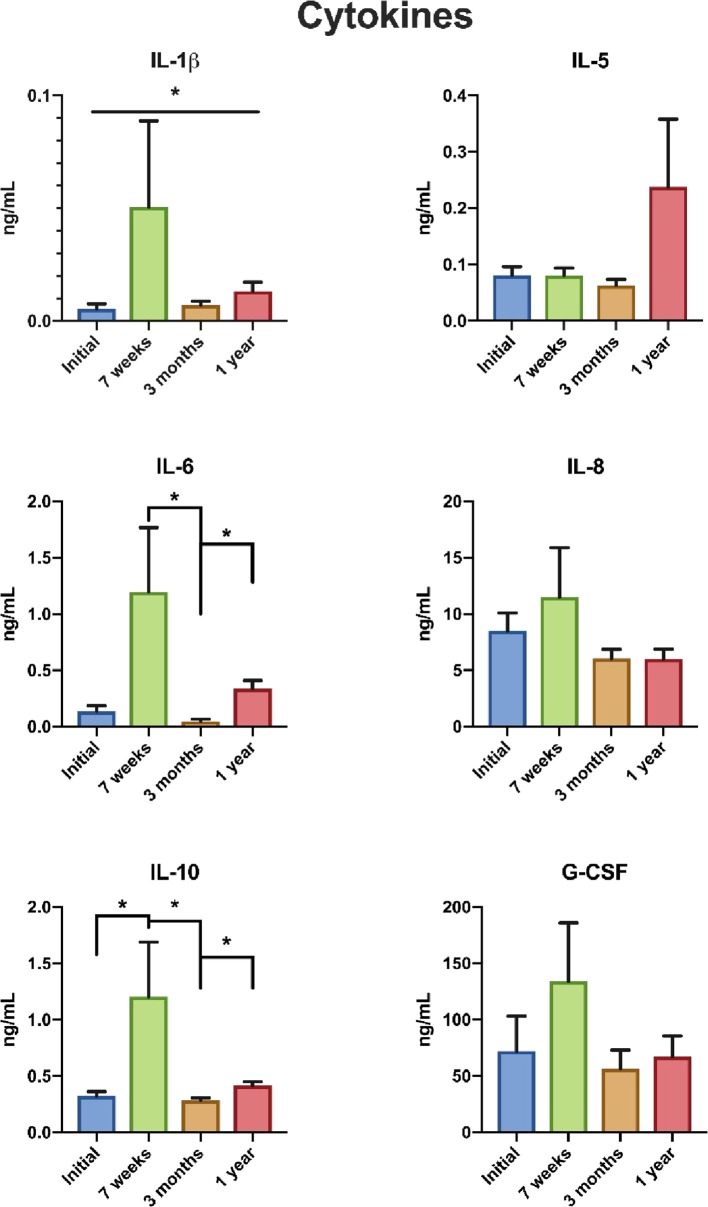
Cytokine levels before treatment, at 7 weeks after start of treatment, 3 months and 1 year after termination of treatment in patients with head and neck cancer. *Statistically significant. G-CSF, granulocyte colony stimulating factor; Il-1β, interleukin 1 beta; Il-5, interleukin 5; Il-6, interleukin 6; Il-8, interleukin 8; Il-10, interleukin 10.

For the whole cohort, an elevation in the levels of IL-1β, IL-6, and IL-10 was noted at 7 weeks after the start of treatment with a return to pre-treatment levels at 3 months (Figure 1) after the termination of treatment. At 1 year after termination of treatment IL-1β and IL-10 levels had elevated slightly again. Levels of IL-1β, IL-6, and IL-10 for the five different treatment modalities are shown in Table 2. When patients were stratified to three treatment groups patients undergoing chemoradiotherapy exhibited an increase of IL-1β, IL-6, and IL-10 at 7 weeks.

**Table 2:** Mean value levels (ng/ml) of Interleukin 1-beta (IL-1β), Interleukin 6 (IL-6), and Interleukin 10 (IL-10) before treatment, 7 weeks after the start of treatment, 3 and 12 months after the termination of treatment in patients with head and neck cancer.

**Treatment**	**Before**	**7 weeks**	**3 months**	**12 months**
**IL-1β**				
CRT	0.0045	0.1523	0.0136	0.0150
CRT+surgery	0.0123	0.0171	0.0014	0.0078
RT+surgery	0.0092	0.0098	0.0059	0.0180
RT	0.0061	0.0085	0.0061	0.0122
Surgery	0.0019	0.0133	0.0028	0.0052
**IL-6**				
CRT	0.2325	3.3606	0.0431	0.3774
CRT+surgery	0.1295	1.4183	0.0769	0.3075
RT+surgery	0.2131	0.4077	0.1307	0.5104
RT	0.0567	0.3296	0.1849	0.1413
Surgery	0.0187	0.0657	0.0466	0.4650
**IL-10**				
CRT	0.3857	3.0287	0.3253	0.4264
CRT+surgery	0.2870	1.5778	0.2715	0.3460
RT+surgery	0.3762	0.5309	0.3224	0.4739
RT	0.2553	0.4822	0.2699	0.3596
Surgery	0.2699	0.2401	0.2367	0.4946

*CRT, chemoradiotherapy; RT, radiotherapy*.

**Figure 2:**
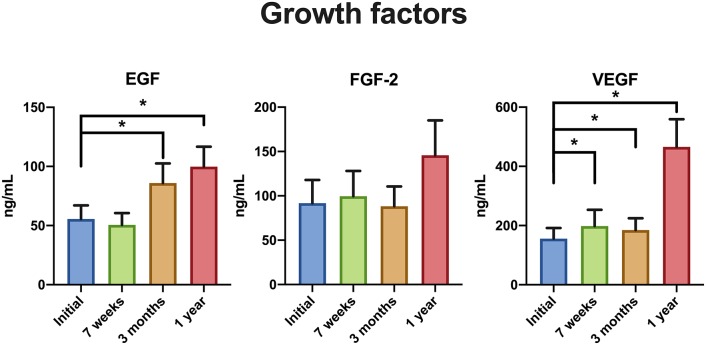
Growth factor levels before treatment, at 7 weeks after start of treatment, 3 months and 1 year after termination of treatment in patients with head and neck cancer. ^*^^,^^**^Statistically significant. EGF, epidermal growth factor; FGF-2, fibroblast growth factor 2; VEGF, vascular endothelial growth factor.

EGF and VEGF levels peaked at 1 year after the termination of treatment (Figure 2).

**Figure 3:**
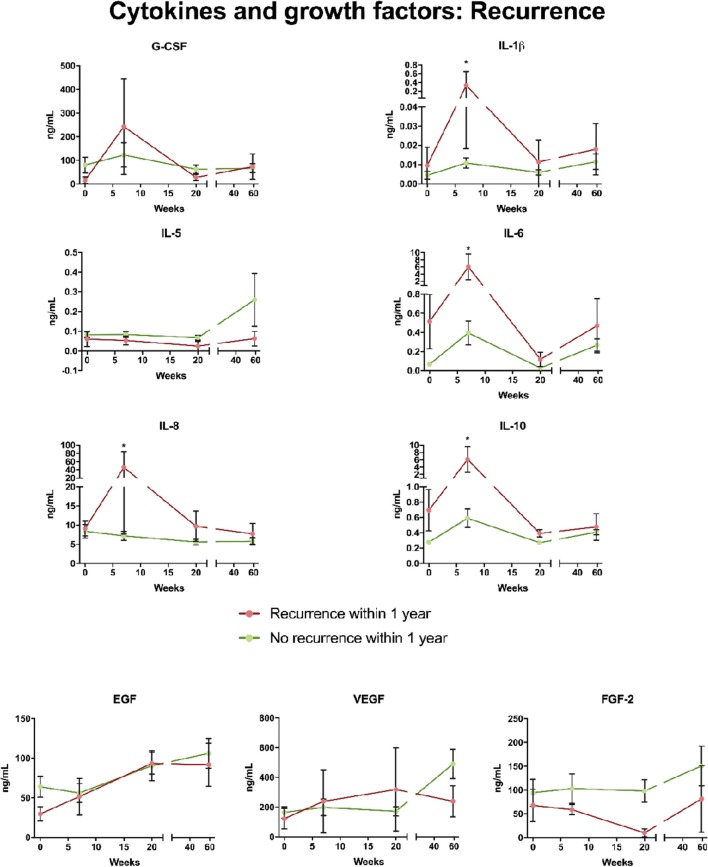
Cytokine and growth factor levels in patients with loco-regional control and in patients with tumor recurrence of head and neck cancer within 1 year after termination of treatment. G-CSF, granulocyte colony stimulating factor; Il-1β, interleukin l beta; Il-5, interleukin 5; Il-6, interleukin 6; Il-8, interleukin 8; Il-10, interleukin 10; EGF, epidermal growth factor; FGF-2, fibroblast growth factor 2; VEGF, vascular endothelial growth factor.

**Figure 4:**
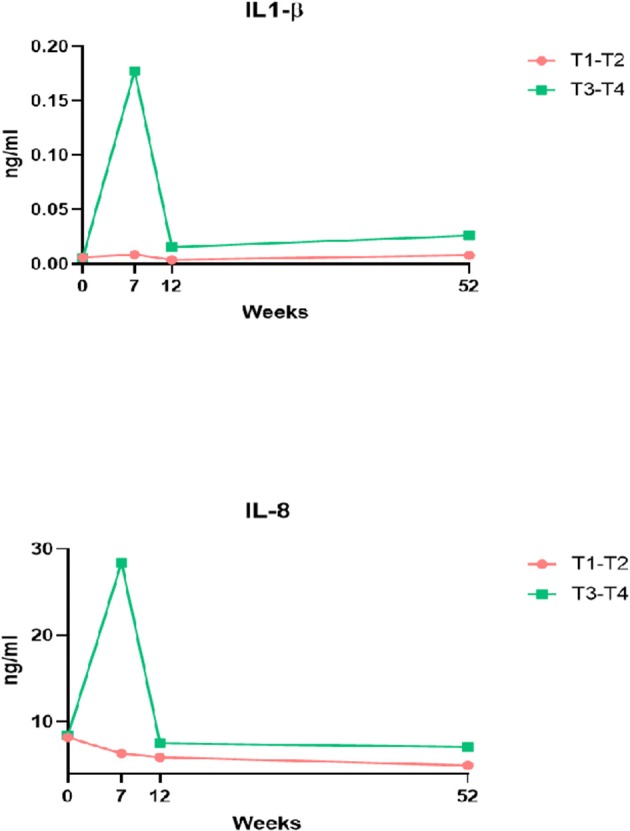
Comparison of levels of IL-1β and IL-8 between patients with T1-T2 and T3-T4 tumors at 7 weeks after start of treatment for head and neck cancer, 3 months and 1 year after termination of treatment. Il-1β, interleukin 1 beta; Il-8, interleukin 8.

Patients with recurrence at 1 year after termination of treatment had higher levels of IL-1β, IL-6, IL-8, and IL-10 at 7 weeks than patients without recurrence as shown in Figure 3. No difference between patients without recurrence and patients with recurrence was noted in cytokine levels pre-treatment and at 3 months and 1 year, respectively. Levels of G-CSM were higher in patients who had a recurrence. Levels of EGF, FGF-2, and VEGF showed no difference between patients with recurrence and disease-free patients (Figure 3). However, a trend that FGF-2 at 3 months was lower in patients with recurrence was observed. When correlated with T stadium patients with T3-T4 had higher levels of IL-1β and IL-8 than patients with T1-T2 measured 7 weeks after the start of treatment (Figure 4).

Cytokines Il-2, Il-4, GM-CSF, and TNF-α were not measurable in any of the patients so no data is shown. IFN-γ was detected in 12 samples of 120 samples in total and is not included in figures and statistics.

The grade of mucositis was documented according to the WHO grading system (17). There was no correlation between the severity of mucositis at 7 weeks after the start of treatment and cytokine levels.

BMI did not correlate with cytokine levels.

## Discussion

Inflammation plays a pivotal role in cancer and numerous studies provide evidence that the immune system can inhibit cancer growth as well as exert pro-tumorous influences (18). Earlier interest has been focused on the role of inflammation in cancer initiation, development and progression, whereas recent advances in the development of immunotherapy have switched the interest to disease control and immune response in association to treatment. The immunologic nature of squamous cell cancer in the head and neck region is poorly understood and needs to be better explored. This explorative study was undertaken to increase the understanding of immune response in association with head and neck cancer treatment. By following the concentrations of cytokines in serum a specific pattern was identified related to the treatment.

An unselected cohort of patients was followed by blood collection at four time points up to 1 year after termination of treatment and the longitudinal pattern of cytokines and growth factors released in the blood compartment was studied as a measure of a systemic immune response to treatment. It was found that both immunostimulating and immunosuppressive pro-inflammatory cytokines, perhaps acting competitively, were raised as a response to chemoradiation and radiotherapy whereas in the four patients only undergoing surgery, minor treatment-related changes were observed. It was shown in the chemoradiotherapy group that IL-1β, IL-6, and IL-10 increased significantly during the first 7 weeks after the start of treatment and thereafter returned to pre-treatment levels.

Nine of ten patients receiving chemoradiotherapy or chemoradiotherapy plus surgery were treated with cisplatin. The observed immune mechanism in this longitudinal observation study clearly demonstrates that chemoradiotherapy may induce not only a local treatment effect on the immune system but also effects far outside the irradiated field. Radiation causes DNA damage and cell death and has earlier been shown to stimulate the secretion of pro-inflammatory cytokines such as IL-6 and IL-8 (19). Circulating levels of cytokines before treatment have previously been analyzed in patients with head and neck cancer (8, 20, 21). Pre-treatment IL-6 concentration was found to predict survival (20). An increase of IL-6 has earlier been shown in 15 patients with head and neck cancer by examining repeated blood samples up to end of chemoradiotherapy which is in agreement with the findings in the present study (22). Earlier studies have indicated that IL-6 is strongly associated with the development of oral cancer and there are experimental indications that anti-IL-6 receptor antibody reduces angiogenesis and tumor growth in squamous cell oral carcinoma (23, 24). In a recent study on a cohort of 32 patients with head and neck cancer treated with chemoradiotherapy IL-1β and TNF-α levels decreased 60 days after treatment as compared to the pre-treatment level whereas IL-6 levels increased (25).

The pattern of growth factors in the present study was different from that of cytokines. A general rise in the levels of FGF-2, EGF, and VEGF was noted at 1 year after the end of treatment. Patients with recurrence within 1 year after treatment tended to have lower levels of growth factors. In previous studies, VEGF and EGF have been shown to correlate with worse prognosis in both head and neck cancer and esophageal cancer (26–28). A similar connection was not seen in our material.

The major goal of the treatment of patients is to induce a curation by eradicating the cancer disease. Radiotherapy and chemotherapy are used to cause the induction of tumor cell killing. By the release of antigens from dying tumor cells, activation of the immune response is taking place (29). The results from the cytokine panel assessment in the present study show that especially chemoradiotherapy engages the immune system. Understanding the immune response at a local and systemic level of patients with head and neck cancer is crucial for the improvement of the therapeutic arsenal. Several experimental research groups have studied antitumor immune response induced by cytostatic drugs, above all cisplatin (30). Cisplatin is an extremely reactive, platinum-containing drug, and has a prone affinity to nucleophiles (31). The apoptotic mechanism induced by cisplatin is mediated mainly through DNA damage. Besides the cytotoxic effects, increasing interests have also been directed to its modulation of the immune system in the tumor microenvironment. It was recently reported that cisplatin *in vitro* at lower doses enhances antigen presentation and T-cell killing (32). Panels of cytokines have been studied in other groups of patients with a malignant disease but not up to 1 year after termination of treatment (20, 22, 25, 33, 34).

Another important question that arises is the influence of tumor volume on the cytokine release. Patients with T3-T4 tumors demonstrated the highest concentration of IL-1β and IL-8 at 7 weeks. This observation is consistent with the hypothesis that the observed immune response is related to tumor volume which in turn is associated with the risk of recurrence and worse prognosis.

The strengths of this study include the longitudinal follow-up of cytokines in the context of head and neck cancer treatment which to our knowledge has not been conducted before. Besides the treatment-dependent alterations, the study pinpoints an inter- and intraindividual variability in the studied cytokines during 1 year after treatment. One can expect that during an early phase of treatment the phenomenon of systemic cytokine release might be attributed to activation of the inflammasome (3). The reduction in cytokine levels at 3 months indicates that stimulation of inflammasome is shut down, most probably due to the clearance of antigens produced by cancer.

The main limitation of the present study is the relatively low number of patients. The study cohort is rather heterogeneous with regard to tumor location as is common in head and neck cancer research. A potential confounder may be the most commonly reported toxicity, oropharyngeal mucositeis, that affects the majority of patients undergoing radiotherapy for head and neck cancer. No correlation was seen between the severity of mucositis and cytokines at 7 weeks after the start of treatment, i.e., at the termination of radiotherapy or chemoradiation when mucositis reached its peak level. The majority of patients in this cohort had a maximum mucositis score of 1–2 (WHO grading system) which is relatively mild but in accord with the lower toxicity seen with intensity modulated radiotherapy (IMRT). Meirovits et al. (22) have previously shown in a cohort of 15 patients that severe mucositis correlates with elevated IL-6 levels during RT, however, 13 of the patients received 2D or 3D conformal technique radiotherapy, which is known to induce more toxicity to the oropharyngeal mucosa than IMRT. It seems unlikely that the observed low-to-medium grade mucositis seen in the majority of patients in the present study contributed to the rise in cytokine levels.

Systemic inflammation seems to be tightly knit to the tumor microenvironment but levels of circulating cytokines and growth factors in peripheral blood do not necessarily reflect their concentration in and around a tumor (35, 36). The study raises a number of questions and a larger study, specifically including more patients receiving radiotherapy or chemoradiotherapy, is warranted to further evaluate the inflammation caused by the combined treatment of ionizing radiation and cisplatin. We will consequently expand our study on cytokines and growth factors to a larger cohort.

To conclude, the observed immune response in this explorative study demonstrates that chemoradiotherapy may induce not only a local treatment effect on the immune system but also effects far outside the irradiated field. Patients that received chemoradiotherapy demonstrated a stronger immune response compared to other treatment groups. The findings of the study indicate that the analysis of a pro-inflammatory panel of cytokines in serum at 7 weeks after the start of treatment could be of prognostic value in patients with head and neck cancer. Our results do encourage further investigation of the relationship between inflammation, treatment modalities, and tumor recurrence. If these findings can be repeated in a larger cohort this could help us identify patients at larger risk for recurrent disease with measurements of pro-inflammatory cytokines under and after treatment. In addition, a larger study could provide clues to potential targets for radiosensitization and immunotherapy. Analyzing tumor biopsies with regards to cytokines could add further value to such a study.

## Data Availability Statement

The datasets generated for this study are available on request to the corresponding author.

## Ethics Statement

The studies involving human participants were reviewed and approved by The Regional Ethical Review Board in Uppsala. The patients/participants provided their written informed consent to participate in this study.

## Author Contributions

GL and YE conceived the study design and primary hypothesis. GL and TA were responsible for patient recruitment. YE organized database and gathering of blood samples. FS and DB performed blood sample analysis. FS was responsible for statistical calculations. All authors discussed the results and contributed to the final manuscript.

### Conflict of Interest

The authors declare that the research was conducted in the absence of any commercial or financial relationships that could be construed as a potential conflict of interest.
